# Cross-cultural adaptation, reliability, and validity of a Chinese version of the swallowing impairment score

**DOI:** 10.3389/fpsyg.2025.1502327

**Published:** 2025-04-09

**Authors:** Weiwei Xu, Yuqiu Zhou, Yuxia Fan, Man Jiang, Guihua Li, Hui Guo, Qing Yang, Dingfen Zeng

**Affiliations:** ^1^Department of Head and Neck Surgery, Sichuan Clinical Research Center for Cancer, Sichuan Cancer Center, Sichuan Cancer Hospital & Institute, University of Electronic Science and Technology of China, Chengdu, China; ^2^Department of Nursing, Sichuan Clinical Research Center for Cancer, Sichuan Cancer Center, Sichuan Cancer Hospital & Institute, University of Electronic Science and Technology of China, Chengdu, China

**Keywords:** swallowing impairment score, thyroidectomy, cross-cultural adaptation, reliability, validity

## Abstract

**Introduction:**

The impact of thyroidectomy on swallowing is prevalent. Difficulties in swallowing can lead to malnutrition, destress and a decline in quality of life. The Swallowing Impairment Score (SIS-6) is a uniquely self-evaluation questionnaire aimed at comprehensively assessing the swallowing impairment status in patients after thyroidectomy. However, there is currently no Chinese version available for use among Chinese populations. The objective of this research is to culturally modify the SIS-6 to a Mandarin Chinese version and validate its psychometric features.

**Materials and methods:**

Initially, the SIS-6 was translated and refined; 30 patients who underwent thyroidectomy were enrolled for the cognitive testing. Subsequently, a total of 468 patients who had undergone thyroidectomy were enrolled to evaluate the psychometric properties of the Chinese version.

**Results:**

The Chinese version of SIS-6 was developed through translation and cultural adaptation processes. No floor or ceiling effects were observed. The scale-level Cronbach's α coefficient was 0.790 with each item ranging from 0.404 to 0.665. The scale-level intra-class correlation coefficient was 0.889 with each item ranging from 0.594 to 0.920. The item-level content validity index ranging from 0.880 to 1, with scale-level content validity index of 0.910. The confirmatory factor analysis verified the two-factor structure of the Chinese version of SIS-6 with factor loadings for each item ranging from 0.530 to 0.810.

**Conclusion:**

Although the Mandarin Chinese version of SIS-6 exhibited gender imbalance within its sample size and lacked a cut-off value, it demonstrated satisfactory psychometric properties overall and served as an effective and reliable tool for assessing swallowing difficulties in patients after thyroidectomy.

## Introduction

Thyroidectomy is the most frequently performed surgical procedure on endocrine glands, and its impact on swallowing is prevalent, even among some patients who do not exhibit confirmed complications (Lombardi et al., 2009; Galluzzi and Garavello, 2019). This symptom can be primarily attributed to several factors, including surgical techniques, laryngotracheal fixation, scar retraction and the possibility of damaging to the external branch of the superior laryngeal nerve or recurrent laryngeal nerve (de Pedro Netto et al., 2006). It has been noted that the frequency of complaints regarding swallowing parallels that of voice-related complaints when patients are queried about these issues. Clinicians may overlook these symptoms for an extended duration as they are often deemed self-limiting and do not consistently align with objective findings (Park et al., 2021). Difficulties in swallowing can increase the risk of malnutrition and dehydration (Limpuangthip et al., 2022), potentially leading to distress in individuals who may experience a decline in their quality of life (Patton et al., 2023).

In China, a significant number of patients undergo surgery for thyroid nodules and cancer each year (Gao and Liu, 2025). For those experiencing swallowing difficulties, the general assessments currently lack sensitivity and encompass a broad range of content, which may not accurately reflect the specific circumstances of this target population (Danić-HadŽibegović et al., 2020). The Swallowing Impairment Score (SIS-6) is a uniquely self-evaluation questionnaire aimed at comprehensively assessing the swallowing impairment status in patients after thyroidectomy (Lombardi et al., 2006). It comprises 6 straightforward statements scored on a scale ranging from 0 (indicating no swallowing alterations) to 4 (indicating maximum swallowing impairment). Meanwhile, the SIS-6 is the most widely used scale for assessing changes in swallowing following thyroid surgery (Lombardi et al., 2006). Currently, scholars from Iran (Lombardi et al., 2009), Italy (Lee et al., 2018), South Korea (Exarchos et al., 2016), and Greece (Li et al., 2023) have successfully implemented the SIS-6 in their studies with satisfactory results. However, the scale has not been validated in China so that the accuracy of assessment will be influenced by language and cultural differences of patients. This research aims to translate and culturally adapt the SIS-6 into Mandarin Chinese and evaluating its reliability and validity.

## Materials and methods

**Figure 1:**
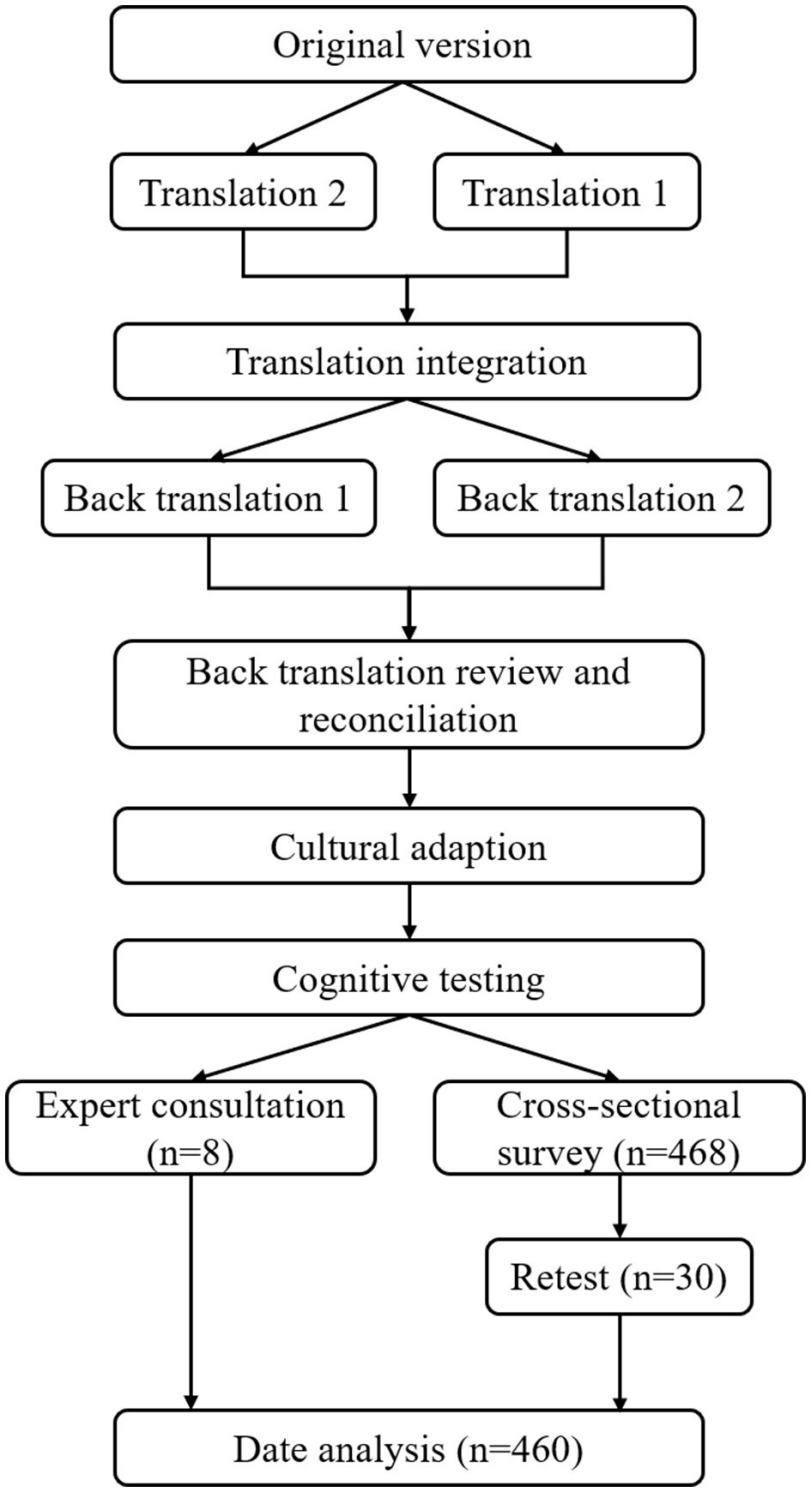
Flow diagram.

The research was split into two components (Figure 1): (1) translation and cross-cultural adaptation; and (2) psychometric validation.

### Phase 1: translation and cultural adaption

After obtaining the original scale, the SIS-6 was translated and culturally adapted into Mandarin Chinese following international guidelines (Beaton et al., 2000; Tsang et al., 2017). The process included the following detailed steps.

#### Step 1: forward translation

The SIS-6 was independently translated into Mandarin Chinese by two researchers who were proficient in both English and their native Chinese language. Translator 1 is a clinical medicine expert who passed CET-6 and studied abroad, ensuring equivalent clinically appropriate translation (FT1). Translator 2 is an English master's degree student with no medical background, ensuring accurate linguistic translation (FT2).

#### Step 2: reconciliation

Postgraduate students specializing in nursing, who are native Chinese speakers and possess a high level of proficiency in English, conducted a comparative and validation study on the two Mandarin Chinese translations of the SIS-6 (FT1 and FT2). Following an analysis of the discrepancies observed among the translators, a synthesized translation (FT12) was generated.

#### Step 3: back translation

The back translation process was completed by two nursing PhDs. Both researchers had significant studying and working experience in head and neck surgery but were unfamiliar with the original scale of the SIS-6. Two English versions (BT1 and BT2) were created after the completion of the back translations.

#### Step 4: back translation review and reconciliation

To detect and address any inconsistencies in translation among different editions, a reconciliation meeting was held between the project leader and all the translators. In the event of significant disparities among the items, the developer would provide repeated explanations until all translated versions were aligned. Subsequently, a prefinal version A was generated after the aforementioned meeting.

#### Step 5: cultural adaption

The cultural adaptation was conducted to assemble the viewpoints and evaluations from different experts on semantic similarity and cultural relevance via email or paper documents. This process involved the participation of five professionals, including 2 experts in thyroid disease nursing, 1 expert in linguistics, 1 expert in nursing education, and 1 thyroid disease expert who has extensive learning experience in Italy, thus having a comprehensive understanding of the Italian language and culture. the average work experience of these experts was 8–24 years (14.40 ± 7.13 years); the degree distribution was as follows: two doctors, two master's degrees, and 1 bachelor's degree; the title distribution was as follows: two professors, two associate professors, and one intermediate title. The original version of the SIS-6, the prefinal version A, and other translated version resources were sent to 5 experts to estimate semantic equivalence and cultural applicability of the translated version A. In addition, they were obligated to offer thorough recommendations for any incorrect translations. Gathering these viewpoints from specialists can aid in enhancing the precision and comprehensibility of each item to generate a prefinal version B. Throughout the phase of cultural adjustment, we maintained regular communication with the developer of the SIS-6 via email.

#### Step 6: cognitive testing

To evaluate the comprehensibility of translation version B and the semantic content of the items, a cognitive assessment was administered as the final stage. The cognitive testing utilized a convenience sample of 30 patients who underwent thyroidectomy from the department of head and neck surgery of Sichuan Cancer Hospital & Institute in Chengdu, Sichuan Province. This hospital is the largest national tertiary level class A cancer hospital in southwest China. The inclusion criteria were as follows: (1) Patients who underwent thyroidectomy; (2) those who gave consent to participate in this study. The exclusion criteria included: (1) patients who had a history of swallowing difficulties or neck surgery; and (2) those who had diseases unrelated to the thyroid gland. Each participant was asked to elucidate the meaning of each item in the scale and report any difficulties encountered in understanding the content. Additionally, participants were instructed to reflect upon the relevance of each element in the revised version B to their post-thyroidectomy condition. After completing the steps, a Mandarin Chinese version of the SIS-6 was formed.

### Phase 2: psychometric evaluation

Psychometric properties of the SIS-6 Mandarin Chinese version include validity and reliability. After obtaining the ethical approval from the Sichuan Cancer Hospital & Institute Institutional Review Board (No.SCCHEC-03-2018-014), the psychometric assessment was split into two components: expert consultations to assess content validity and cross-sectional survey to assess construct validity, internal consistency reliability, and test-retest reliability.

### Expert consultation

To ascertain the content validity, a group of experts was contacted through email or paper documents. These experts were required to fulfill certain criteria: (1) possess substantial knowledge in the fields of head and neck surgery or cross-cultural translation methodology, (2) possess a minimum of 10 years of professional experience, and (3) express willingness to partake in the research. A total of eight proficient professionals were enlisted to evaluate the pertinence of each item to the measurement objective, utilizing a Likert-scale consisting of four points, ranging from 1 (not related at all) to 4 (totally related).

### Cross-sectional survey

#### Participants

This survey utilized a convenience sampling method. All the participants originated from the department of head and neck surgery of Sichuan Cancer Hospital & Institute. To evaluate the psychometric properties, the sample size should be 10–20 times the number of items (Maguire et al., 2023). Previous studies have indicated that a suitable sample size for conducting confirmatory factor analysis (CFA) is approximately 300 (Alam et al., 2022). The inclusion criteria were as follows: (1) Patients who underwent thyroidectomy; (2) those who gave consent to participate in this study. The exclusion criteria included: (1) patients who had a history of dysphagia or neck surgery; and (2) those who had diseases unrelated to the thyroid gland.

#### Data collection and analysis

We have conducted an online data collection and paper document collection method for both convenience and safety reasons. From January to March 2023, potential eligible participants were sent website links or paper questionnaires. The questionnaire consists of three parts: (1) An explanation and informed consent form that explains the study's purpose, anonymity principle, and voluntary participation; (2) Demographic characteristics such as gender, age, educational level, preoperative diagnosis, staging of tumor, and type of thyroid surgery; and (3) The Mandarin Chinese version of the SIS-6. Data analysis was conducted using SPSS4.0 and AMOS 24.0 software. Demographic characteristics were presented by frequencies and percentages (%), while mean and standard deviation were used to present the scores of the SIS-6.

#### Floor and ceiling effects

Floor and ceiling effects means were evaluated by the percentage of participants obtaining the highest and lowest possible scores on the SIS-6. The absence of floor and ceiling effects was determined if fewer than 20% of the participants attained the lowest or highest scores.

#### Internal consistency reliability

Cronbach's α coefficients and item-total correlation were utilized to establish internal consistency reliability. If Cronbach's α coefficients were above 0.70 and item-total correlation with a value >0.3, it would be deemed acceptable.

#### Test–retest reliability

Spearman's Rho correlation analysis was used to evaluate the intra-class correlation coefficient (ICC), which was categorized as poor (< 0.50), moderate (between 0.50 and 0.75), good (between 0.75 and 0.90), and excellent (over 0.90) (Tavakol and Dennick, 2011).

### Content validity

The calculation of the content validity index (CVI) was conducted at both the item level (I-CVI) and scale level (S-CVI) using the scores provided by experts. A four-point Likert scale was employed to assess the I-CVI, specifically focusing on the ratings of experts who deemed the item as three (related) or four (totally related). The S-CVI was obtained by averaging the I-CVIs of all items. It is recommended that an I-CVI of 0.78 (Polit et al., 2007) and an S-CVI of 0.90 or higher indicate sufficient levels of content validity (Ma et al., 2021).

### Construct validity

To assess the construct validity, both an exploratory factor analysis (EFA)and CFA were conducted, a sample of 460 cases was divided into two groups by SPSS software, one for EFA and one for CFA, In terms of their characteristics, both groups exhibited similarities. A Kaiser-Meyer-Olkin (KMO) value higher than 0.60 and Bartlett's spherical test yielded < 0.05 were deemed suitable criteria for conducting factor analysis on the data (Munin et al., 2016). Principal component analysis (PCA) and maximum variance rotation were used to conduct EFA. Items with a loading of >0.40 and the factors that emerged from EFA explain more than 50% of the scale were considered as contributing to a factor and retained (Cabrera-Nguyen, 2010). We retained factors using multiple criteria including eigenvalues >1.0, eigenvalues greater than the point of change in the slope of decreasing eigenvalues as observed in a scree plot, parallel analysis, number of items loading, and understanding of the theoretical expectations (Barnes, 2021). Next, according to the results of EFA, the CFA was used to assess the suitability between the established models and the data, The following indices were used to evaluate the goodness-of-fit of the model and the data: ratio of chi-square and degree of freedom (χ2/df), goodness-of-fit index (GFI), Tucker-Lewis index (TLI), comparative fit index (CFI), and root mean square error of approximation (RMSEA). the model was considered acceptable when χ^2^/df < 3.0, CFI, GFI, and TLI were 0.90 or higher, RMSEA < 0.08, and SRMR < 0.05 (Wolf and McNeish, 2023; Bentler and Bonett, 1980).

## Results

### Translation and cultural adaption

Discrepancies were primarily observed in Step 2 (Reconciliation) and Step 5 (Cultural adaptation). The initial concern pertained to the interpretation of “bolus transit” in items 3 and 4. One translator failed to convey the food's attributes, focusing solely on the act of swallowing. Conversely, another translator rendered it as “solid” passing through the throat, emphasizing the influence of food characteristics on the swallowing process. After conducting a harmonization meeting and consulting with the developer, we have reached a consensus that the texture of food plays a significant role in the sensation of swallowing, thereby emphasizing the significance of stickiness as a characteristic of food. Additionally, in the process of cultural adaptation, it has been suggested by two experts that emphasis should be placed on the severity of difficulty in swallowing (item 1) and in swallowing fluids (item 6), aligning with the original version. After careful consideration, it was unanimously agreed upon that the degree of swallowing difficulty should be accurately conveyed in the translation. Moreover, one expert raised concerns regarding the potential confusion between item 2 and item 5. After expert collective discussion and consideration of the clinical situation, it was determined that item 2 is associated with the perception of food not traversing the throat smoothly, while item 5 pertains to the sensation of foreign matter in the throat. Consequently, to elucidate the distinction between these two phenomena, we have incorporated scenarios such as “Food does not pass smoothly through the throat.” No other disagreements were identified about the Mandarin Chinese version of SIS-6. All 30 participants in the cognitive assessment unanimously agreed that the items were effectively articulated and readily comprehensible. The cognitive testing outcomes did not necessitate any modifications. All the SIS-6 items of both the English version and the final Mandarin Chinese version are listed in Table 1.

**Table 1:** The items of the SIS-6 in both English version and Chinese version.

**Items**	**English version**	**Chinese version**
1	I use a great effort to swallow.	我吞咽很费力
2	I feel a throat obstacle during swallowing.	吞咽时,我感觉喉咙有梗阻感(食物通过咽喉不顺畅)
3	I feel pharyngeal annoyance during bolus transit.	吞咽固体时,我感觉咽喉部不适
4	I cough during bolus transit.	吞咽固体时,我会出现呛咳
5	I feel sensation of foreign body in pharynx.	我的咽喉部有异物感
6	I have some difficulties for fluid swallowing.	我吞咽液体有些困难

### Psychometric evaluation

#### Demographic characteristics

We received 468 completed questionnaires, of which 460 were valid, resulting in a response rate of 98.29%. Female participants accounted for 74.30% (342) of the total. Detailed demographic characteristics of the participants are listed in Table 2.

**Table 2:** Demographic characteristics of participants (*n* = 460).

**Variable**	***N***	**%**
**Gender**		
Male	118	25.70
Female	342	74.30
**Length of hospital(day)**		
≤7	337	73.30
>7	123	26.70
**Age (years)**		
≤18	7	1.50
>18	453	98.50
**Education**		
Primary school or less	74	16.10
Middle school	126	27.40
High school or more	260	56.50
**Disease diagnosis**		
Thyroid cancer	413	89.80
Thyroid nodule	45	9.80
Goiter	2	0.40
**Surgical approach**		
Open surgery	384	83.50
Laparoscopes or Robotics	73	15.80
Transoral approach	3	0.70
**Type of medical insurance**		
Self-funded	89	19.35
Medical insurance for urban employees	225	48.91
Medical insurance for urban residents	102	22.17
Rural cooperative medical scheme	44	9.57
**Professional status**		
Unemployed	61	13.26
In employment	386	83.91
Retirement	13	2.83
**Monthly per capita household income (Yuan)**		
≤3000	36	7.83
>3000	114	24.78
>5000	234	50.87
>7000	76	16.52

#### Floor and ceiling effects

The scores for each item of the SIS-6 are listed in Table 3. The lowest (0) and highest (Bentler and Bonett, 1980) scores were achieved by only 3.00% and 0.90% of the participants, respectively. Thus, neither floor nor ceiling effects existed.

**Table 3:** The item scores and corrected item–total correlation coefficients (*n* = 460).

**Items**	**Mean**	**SD**	**Corrected item–total correlation coefficients**	**Cronbach's alpha, if an item deleted**
1. I use a great effort to swallow	2.73	1.28	0.66^**^	0.73
2. I feel a throat obstacle during swallowing	2.55	1.45	0.62^**^	0.74
3. I feel pharyngeal annoyance during bolus transit	2.92	1.31	0.67^**^	0.73
4. I cough during bolus transit	1.21	1.16	0.40^**^	0.78
5. I feelsensation of foreign body in pharynx.	2.28	1.58	0.48^**^	0.78
6. I have some difficulties for fluid swallowing	1.35	1.29	0.45^**^	0.78

SD, standard deviation;

** *P* < 0.01.

#### Internal consistency reliability

Internal consistency reliability for the whole scale was 0.79. All the corrected item-total correlation coefficients ranged from 0.40 to 0.67. The Cronbach's α value of the SIS-6 ranged from 0.73 to 0.78 after removing each item, but it did not exceed the Cronbach's α value of the scale (Table 3).

#### Test–retest reliability

To determine the test-retest reliability of the Mandarin Chinese version of SIS-6, the identical participants who voluntarily took part in the subsequent survey were administered the questionnaire. This resulted in the collection of 30 usable questionnaires. The test-retest reliability surveys were conducted with an approximate interval of 2 weeks. The ICC for the scale was 0.89, and the ICCs for each item ranged from 0.59 to 0.92. Detailed results are listed in Table 4.

**Table 4:** Test–retest reliability of the Chinese version of SIS-6 for the total score, and single items.

	**Test score mean (SD)**	**Retest score mean (SD)**	**ICC (95% CI)**	***P* value**
Total score	14.23 (7.38)	12.68 (5.59)	0.89 (0.78–0.95)	<0.001
1. I use a great effort to swallow	2.67 (1.42)	2.33 (1.12)	0.59 (0.28–0.85)	<0.001
2. I feel a throat obstacle during swallowing	2.73 (1.48)	2.37 (1.25)	0.75 (0.54–0.87)	<0.001
3. I feel pharyngeal annoyance during bolus transit	2.70 (1.49)	2.53 (1.46)	0.86 (0.72–0.93)	<0.001
4. I cough during bolus transit	2.20 (1.35)	1.97 (1.16)	0.92 (0.84–0.96)	<0.001
5. I feel sensation of foreign body in pharynx.	2.43 (1.81)	2.17 (1.67)	0.78 (0.60–0.89)	<0.001
6. I have some difficulties for fluid swallowing	1.50 (1.41)	1.30 (1.26)	0.82 (0.65–0.91)	<0.001

SD, standard deviation; ICC, Intraclass correlation coefficient.

#### Content validity

Regarding content validity, the consensus among experts was that all items were related or totally related to the measurement purpose of the SIS-6. The values of I-CVI ranged from 0.88 to 1, while the S-CVI was determined to be 0.91.

#### Construct validity

**Figure 2:**
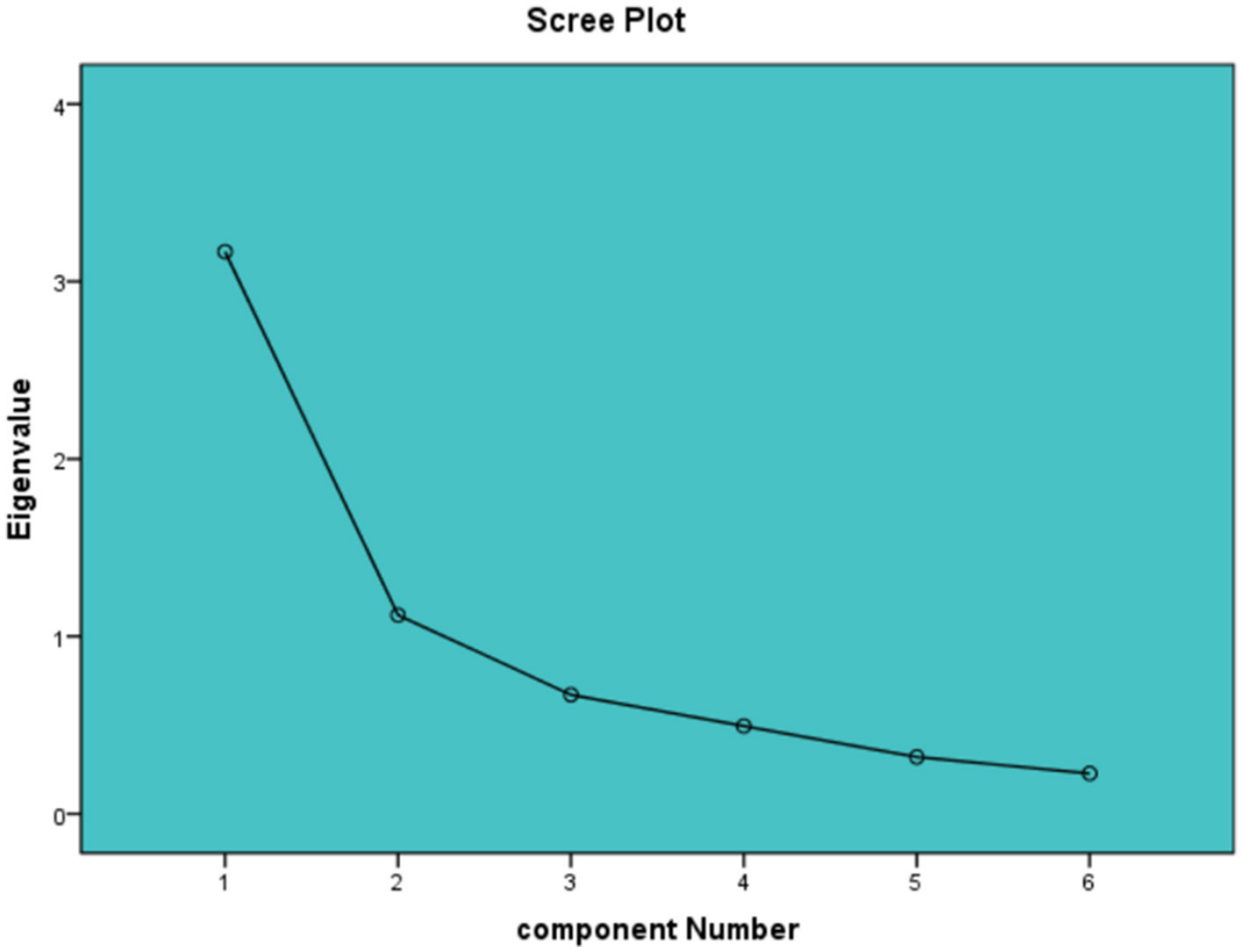
Scree plot of SIS-6 (*n* = 139).

**Figure 3:**
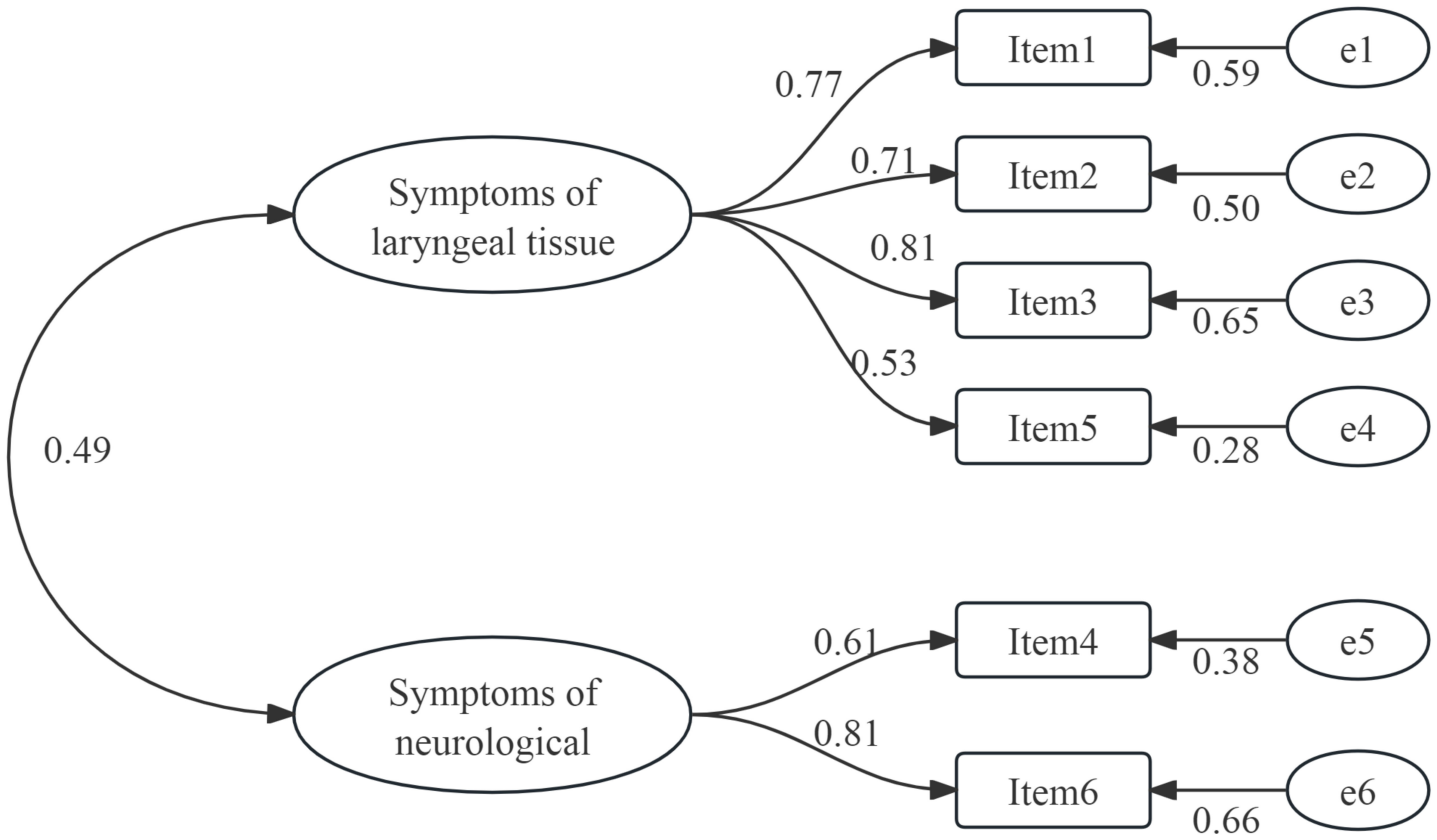
Confirmatory factor analysis (*n* = 321).

A total of 460 samples were partitioned into two distinct subsets, with one subset consisting of 139 samples designated for EFA, and the other subset comprising 321 samples allocated for CFA. After conducting EFA, it was found that the KMO value was 0.80 and Bartlett's spherical test resulted in *X*^2^ = 332.46 (*p* < 0.01), indicating that the data was appropriate for factor analysis. The study yielded two prominent factors (eigenvalues > 1), which collectively accounted for a cumulative variance of 71.46%. The scree plot (Figure 2) confirmed the validity of this outcome. Moreover, all items demonstrated factor loadings ranging from 0.64 to 0.89, surpassing the threshold of 0.40, and no instances of cross-loading were observed. The two common factors indicated are shown in Table 5. To provide additional validation for the adequacy of the two-factor model's structural fitting, CFA was utilized. Parameter values for model fit were *X*^2^/df = 1.19, CFI = 0.99, GFI = 0.99, TLI = 0.99, RMSEA = 0.02, and SRMR = 0.02. The factor loadings between the items and corresponding latent variables ranged from 0.53 to 0.81, as shown in Figure 3.

**Table 5:** Factor loadings of the SIS-6 (*n* = 139).

**Factor**	**Items**	**Factor loading**	**Eigenvalue**	**% of variance**
Symptoms of laryngeal tissue	1. I use a great effort to swallow	0.84	2.73	52.79
	2. I feel a throat obstacle during swallowing	0.89		
	3. I feel pharyngeal annoyance during bolus transit	0.87		
	5. I feel sensation of foreign body in pharynx.	0.64		
Symptoms of neurological	4. I cough during bolus transit	0.85	1.56	18.67
	6. I have some difficulties for fluid swallowing	0.85		
Total variance explained (%)				71.46

Factor loadings ≥ 0.4 were considered acceptable.

## Discussion

Post-thyroidectomy swallowing impairment is a common issue that significantly reduces patients' quality of life over an extended period. To enhance the management of symptoms associated with postoperative swallowing impairment, we undertook a cultural adaptation and translation of the SIS-6 into Mandarin Chinese, followed by a comprehensive evaluation of the psychometric properties of this version. This Mandarin Chinese version of the adapted version has been well-received by patients who have undergone thyroidectomy, especially those who have had thyroid cancer surgery, and has proven its reliability and effectiveness. It is a valuable tool for clinical professionals to assess the severity of swallowing symptom after thyroidectomy.

During the process of cultural adaptation, we adhered to the revised Brislin translation principles and held regular expert meetings to address any challenges related to achieving precise comprehension. To ensure cultural relevance, an expert in Italian culture was invited to assess the language expression habits and semantic equivalence of the scale. A thorough understanding of the original cultural context and the significance proved highly beneficial. Following a comprehensive reconciliation meeting and incorporating recommendations from the original developer, we prioritized specific elements of the SIS-6 to align it more effectively with the prevailing patient circumstances. The act of swallowing is notably influenced by the characteristics of food, as substantiated by previous scholarly investigations and our firsthand clinical observations (Otani et al., 2023). Generally speaking, liquid enters the throat from the oral cavity at a relatively rapid velocity, allowing insufficient time for airway closure mechanisms to engage adequately. Conversely, solid food can reduce flow rate, thereby providing additional time to prevent airway obstruction- consequently averting coughing and discomfort. In conclusion, we emphasized that when translating item 3, it was essential that food characteristics are represented as solid. To better illustrate the distinctions between item 2 and item 5, we provided an example of item 2. All these changes were obtained from experts and patients, and they all agreed with these scale items.

A total of 30 patients who had undergone thyroidectomy participated in cognitive testing, during which they demonstrated full comprehension of the items and a strong personal connection. Additionally, the Mandarin Chinese version did not display any floor or ceiling effects. These findings indicate that each component of the scale was effectively conveyed, comprehensible, and appropriately articulated.

The results of the internal consistency and test-retest reliability analysis demonstrated that the Mandarin Chinese adaptation of the SIS-6 exhibited congruence with the original version. Specifically, the Cronbach's α coefficient for the SIS-6 was determined to be 0.79, which was deemed acceptable. Additionally, the ICC for the whole scale was 0.89, signifying a stable comprehension of postoperative swallowing challenges. It is noteworthy that item 1′s ICC value did not reach a desirable level, yet it remained acceptable at 0.59, suggesting a reasonable degree of temporal variability in patients' swallowing difficulties. It may be valuable to consider early intervention measures to improve swallowing outcomes. Our findings indicate that the Mandarin Chinese version of the SIS-6 instrument proves to be a reliable mean of evaluating swallowing difficulties, as it consistently measures impairment and demonstrates temporal stability.

As for content validity, all the experts unanimously thought that each item in the SIS-6 could adequately reflect the purpose of the measurement. The I-CVI value varied from 0.88 to 1, and the S-CVI was 0.91. These findings collectively demonstrate the Mandarin Chinese version to possess exceptional content validity. The hypothesis proposed by the original scale designer was validated through the application of EFA, resulting in the identification of two prevalent factors. Each of the prevalent factors displayed factor loadings that exceeded 0.60, and the combined contribution of these two factors accounted for 71.46% of the scale, indicating the strength and stability of the two-factor structure. Meanwhile, CFA verified that the two-factor structure of the SIS-6 had a satisfactory model fit. All the factor loadings in each prevalent factor were above 0.60, which further confirmed the stability of the two-factor structure.

## Limitations and future directions

Some limitations of the present study must be considered. First, the study utilized a convenience sample drawn from hospital in Chendu City, which may not fully capture the diversity of the broader patients undergoing thyroidectomy population. This limitation affects the generalizability of our findings to other regions and cultural settings. To ensure accuracy, it is recommended that multi-center studies should be carried out in the future.

Second, we acknowledge that the disease diagnosis of our sample were heterogeneous, with thyroid cancer being much more frequent (413 participants with thyroid cancer representing 89.80% of the sample) than others. As thyroid cancer is the second most common cancer among women in China, in line with other studies (Gong et al., 2024), there was a significant gender imbalance in our study, with a considerably higher proportion of female participants (74.30% of the sample). This may raises potential questions related to gender disparities in swallowing studies, prior research has indicated that gender plays a significant role in individuals experiencing difficulties with swallowing (Rodriguez et al., 2023). As a result, any gender prejudice in the study group could impact the applicability of the survey. Thus, future studies might prioritize achieving a more balanced representation of gender to attain a comprehensive understanding of the SIS-6.

Furthermore, the SIS-6 measurement lacks a definitive cutoff point. Previous studies have categorized scores as mild-moderate, or severe, while others have used low-high distress categories (Ostovar et al., 2022). These cutoff points are based on the researchers' experiences. Therefore, in the future, statistical methods such as latent profile analysis and quantile methods can be used to determine the cutoff score of this tool.

## Conclusions

The cultural adaptation and translation of the SIS-6 into Chinese demonstrated its reliability and accuracy as an instrument for assessing swallowing difficulties in Chinese patients undergoing thyroidectomy. Although this study has gender imbalance in the sample and lacks a cutoff points for the scale, the validated and innovative resource provides Chinese healthcare professionals and administrators with a means to effectively manage dysphagia in clinical settings, potentially enhancing the quality of life for thyroidectomy patients in the future.

## Data Availability

The original contributions presented in the study are included in the article/supplementary material, further inquiries can be directed to the corresponding author.
